# The Ghosts in the Computer: The Role of Agency and Animacy Attributions in “Ghost Controls”

**DOI:** 10.1371/journal.pone.0026429

**Published:** 2011-11-04

**Authors:** Francys Subiaul, Jennifer Vonk, M. D. Rutherford

**Affiliations:** 1 Department of Speech and Hearing Science, The George Washington University, Washington, D.C., United States of America; 2 Department of Psychology, Oakland University, Rochester, Michigan, United States of America; 3 McMaster University, Hamilton, Canada; Freie Universitaet Berlin, Germany

## Abstract

Three studies evaluated the role of 4-year-old children's agency- and animacy-attributions when learning from a computerized ghost control (GC). In GCs, participants observe events occurring without an apparent agent, as if executed by a “ghost” or unobserved causal forces. Using a touch-screen, children in Experiment 1 responded to three pictures in a specific order under three learning conditions: (i) trial-and-error (Baseline), (ii) imitation and (iii) Ghost Control. Before testing in the GC, children were read one of three scripts that determined agency attributions. Post-test assessments confirmed that all children attributed agency to the computer and learned in all GCs. In Experiment 2, children were not trained on the computer prior to testing, and no scripts were used. Three different GCs, varying in number of agency cues, were used. Children failed to learn in these GCs, yet attributed agency and animacy to the computer. Experiment 3 evaluated whether children could learn from a human model in the absence of training under conditions where the information presented by the model and the computer was either consistent or inconsistent. Children evidenced learning in both of these conditions. Overall, learning in social conditions (Exp. 3) was significantly better than learning in GCs (Exp. 2). These results, together with other published research, suggest that children privilege social over non-social sources of information and are generally more adept at learning novel tasks from a human than from a computer or GC.

## Introduction

A child's environment is replete with information. Some of this information comes from human sources, such as the actions of parents, siblings and strangers. Other sources of information are the many objects and artifacts populating households. Various researchers have shown that young children use statistical regularities to predict the behavior of objects and individuals in their environments [1], [2], [3], [4]. These skills are likely to facilitate children's ability to learn from and imitate human actions as well as the effects of those actions [5], [6], [7], [8], often referred to as emulation [9]. In an effort to decouple both social and asocial influences on vicarious learning and to test different forms of emulation learning, researchers have employed “ghost conditions” (GCs). In the typical GC, children observe a ‘demonstration’ where a target action is executed without a model present, as if done by a ‘ghost’. Despite the fact that no model is present in GCs and, consequently, no ostensive or referential cues are available to children, children as young as 17 months evidence learning by replicating the observed event [10], [11], [12], [13], [14], [15]. Given that GCs are informationally impoverished in comparison to conditions where a live model demonstrates target responses, it is surprising that children learn under such conditions at all.

The “Natural pedagogy” view of child learning cannot account for learning in these contexts because learning is thought to rely on ‘ostensive’ (i.e., referential) or affective cues provided by agents [16]. More contemporary social learning theorists believe that learning in GCs is a more basic form of vicarious learning; one that is not based on social learning *per se*, which is presumed to require inferring the goals of actors, but rather on affordance learning, which involves replicating physical end states [17], [18], [19], [20]. Among developmental psychologists, the performance of children in GCs has been explored variously. Huang and Charman [13], in an extensive exploration of children's observational learning skills, reported that children copied the spatial transformation of a block when executed by a model (i.e., imitation) as well as when the block spatially transformed itself, without a model, in a GC (i.e., presumably emulation). Other studies have used a bi-directional task where participants have to open a box using one of two techniques, for example push versus pull [15] or slide-left versus slide-right [12]. In these studies children older than 24-months of age copied the demonstrated technique in both a full demonstration, with a model present, and in a GC, where events occurred independently. Given that the actions participants had to execute in these and related studies were simple (one-step) actions and already present in participants' behavioral and motor repertoires [10], [12], [14], [15], performance may be explained by emulation [21] or even motor fluency; a type of recognition memory where familiar items activate associated motor schemas [22], [23]. However, such explanations fail to explain learning in GCs that involve complex (multi-step) responses that involve tool-use [24] or hierarchical responses that are arbitrary and novel to the child [25].

Subiaul and colleagues [25] developed a GC using what is arguably the most opaque of human tools, the computer. This computerized paradigm is preferable to more traditional tool-paradigms because it allows for greater stimulus control. Specifically, this paradigm allows researchers to precisely define what is familiar and what is novel to the participant; variables that might differentially affect whether or not children learn in ghost controls. Moreover, the computer paradigm eliminates the need for independent raters to make subjective measures of imitation or learning because the computer automatically controls the presentation of stimuli and records all responses.

**Figure 1:**
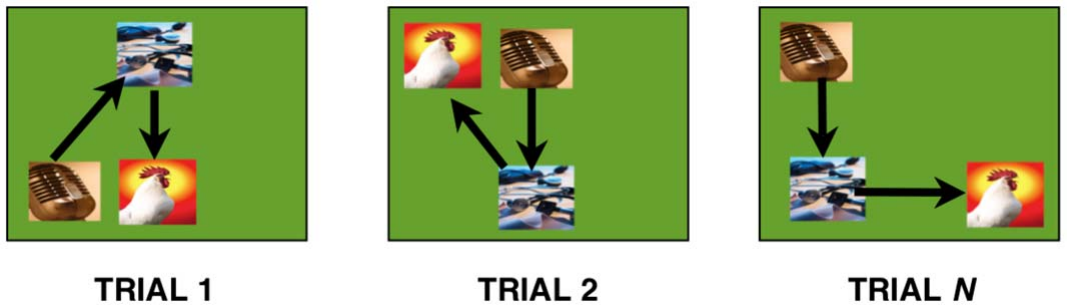
Simultaneous Chaining Paradigm. Arbitrary pictures appear simultaneously on a touch-screen. The task is to touch each picture item in a specific order. From trial to trial, pictures change spatial configuration.

**Figure 2:**
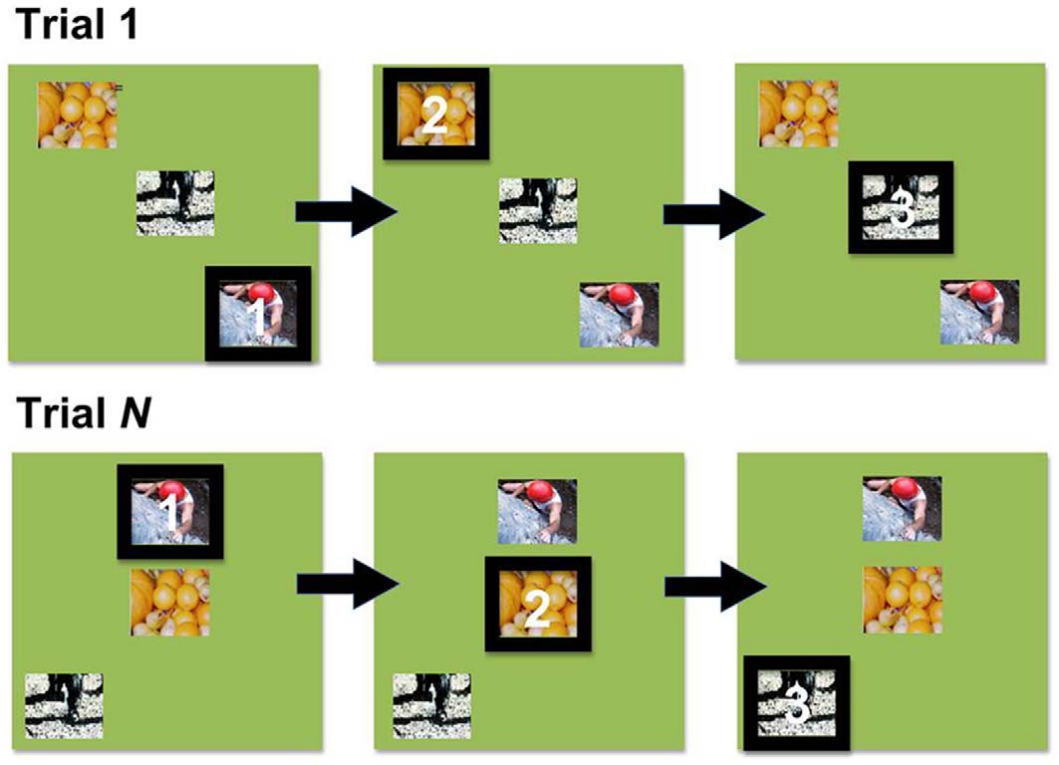
Ghost Control. Using the Simultaneous Chaining Paradigm, the computer automatically highlights with a black border the picture items on the screen in the target order. As in the standard Simultaneous Chaining Paradigm, picture items randomly change spatial configuration from trial to trial and the procedure repeats.

Using this computerized task, Subiaul and colleagues assessed children's ability to learn novel ordinal rules. During Training, three- and four-year olds learned that they had to respond to pictures displayed simultaneously on a touch-screen in a specific order (Figure 1). Following each response, children received social feedback from the model such as, “That's right” and non-social feedback from the computer that included a black border that flashed around a picture when touched in the correct order. Following this Training period, children received two conditions prior to Testing: *Social* and *GC*. In the Social condition, an experimenter demonstrated how to respond to four novel pictures over three consecutive trials before allowing the child to make a response. In the GC, the computer flashed a black border around each picture on the screen in the target order over three consecutive trials before the child was allowed to make a response (Figure 2). Results showed that three- and four-year olds learned significantly above chance levels in both demonstration conditions on the very first trial, and there were no significant differences between Social and GC conditions.

Though using different paradigms, these studies have converged on similar results showing that children of various ages learn in GCs. In some cases, children learn as much in GCs as in social conditions with live models [e.g., 25]. As a result, researchers have concluded that emulation and affordance learning plays a role in children's social learning abilities. However, such terms say little as to exactly *how* learning is achieved under such impoverished circumstances; particularly when what is being learned is entirely new to the participant and cannot be achieved by simply copying the end result of an action. We consider two alternatives: the non-mentalistic, causal “Emulation Hypothesis”, and the mentalistic, social “Agency Attribution Hypothesis.”

At one extreme, Byrne [26] argues that emulation includes all instances of vicarious learning that do not include the copying of specific actions. As a result, any learning that occurs in GCs or doesn't involve copying motor behavior is considered emulation learning. This expansive definition of emulation is problematic because it excludes certain forms of high-fidelity copying that do not involve copying motor actions *per se* such as vocal [27] and cognitive imitation [28]. At the other extreme, Subiaul and colleagues [25] have advanced the hypothesis that children learn in GCs by attributing specific goals to the inanimate object(s) behaving in a goal directed fashion in the context of GCs. In this sense, children would learn in GCs much as some have argued that they learn from human models [29], [30]. That is, by appropriating the goals and actions of the model. The agency-attribution hypothesis is motivated by research showing that humans of all ages have a perceptual bias that results in attributions of agency and animacy to inanimate objects that share one or more of the following cues: self-propelled, goal-directed and varied trajectories [adults: 31], [ infants: 32], [33], [34], [35]. Given that GCs have many of these features, children may either attribute agency or animacy to the computer itself or infer the existence of an unseen agent whose goals are causally related to the action. Whereas several classic studies in developmental psychology have suggested that infants do not learn from mechanical or inanimate objects [36], [37], Biro and Leslie [38] have demonstrated that the addition of various cues associated with agency and animacy reverse these results.

Below, we present a series of studies that systematically test this agency-attribution hypothesis using a complex GC where simple end-state emulation learning explanations do not apply and learning is optimally achieved through high fidelity copying (or imitation) mechanisms [25], [39]. Our prediction is that agency-attribution and features associated with agency and animacy should positively affect learning in GCs. If the agency-attribution hypothesis is correct, then there should be a relationship between perceived agency and first trial accuracy.

In a pilot study, Subiaul and Vonk [40] manipulated four-year-old children's perception of the computer prior to testing them in a computerized GC. For example, children in an Agency-Attribution condition were told that the computer “is alive” and “like you and me.” Children in a Mechanical Attribution condition were told that the computer was “just a machine” and “it doesn't matter what the computer does.” A third group of children in a No Attribution condition were simply instructed to “Watch the computer.” Consistent with the Agency Attribution Hypothesis [25], children in the Agency Attribution condition learned at levels significantly above chance. Children in the Mechanical Attribution condition did not. However, the script may have biased the results in unintended ways. For instance, in the Agency Attribution condition children were told that the computer was “like you and me,” potentially triggering a ‘like-me’ mechanism that has been implicated in imitation learning [41]. And, telling children in the Mechanical Attribution that, “it doesn't matter what the computer does,” may have suppressed attention and learning, independently of notions of agency and animacy. Here we replicated the pilot experiment, correcting for these possible confounds.

Experiment 1 replicated the methods of Subiaul and Vonk [40] with a larger sample of children, using a simplified version of the simultaneous chaining task that included three rather than four-picture lists, a shorter Agency-, Mechanical- and No-Attribution script as well as a short (10 question) survey assessing children's agency and animacy attributions following testing (cf., Methods and Survey S1). All of the children were familiarized with the task prior to testing. Following Training, four-year olds were tested in three conditions: (a) baseline, trial-and-error learning, (b) social, where a model demonstrated the correct response, and (c) one of three randomly assigned GCs, modeled after those described above: (a) No Attribution, (b) Mechanical-Attribution, or (c) Agency-Attribution. During GCs the computer acted as a model, highlighting pictures with a black border and a chime in the target serial order (cf., Figure 2). Prior to testing in each of the GCs, the model read children a simple script that attributed either agency to the computer (Agency-Attribution), mechanical, inanimate attributions (Mechanical-Attribution), or no attributions (No Attribution) [cf., Methods]. The Agency-Attribution Hypothesis predicts that children will spontaneously attribute agency to the computer in the GC and that these attributions will positively correlate with learning on the very first trial.

## Experiment 1

### Results and Discussion

#### First Trial Accuracy

Binomial tests were used to compare the probability of being correct on the first trial in Baseline, Social and the three Ghost conditions to chance performance (*p* = .165). Results revealed that while all children were at chance in the Baseline condition (*p*>.50), all children were significantly above chance in all three of the GCs (*p's*<.01) as well as in the Social (*p*<.001) condition; replicating results reported by Subiaul and colleagues for three- and four-year olds [25]. Table 1 provides a summary of the results.

**Table 1:** Summary of results in Experiments 1–3.

EXPERIMENT 1	N	Baseline	Social	Ghost	Survey	Correlation
No Attribution	18	0.06	0.67	0.50	3.44	0.08
Agency Attribution	14	0.21	0.57	0.43	4.00	0.44
Mechanical Attribution	17	0.39	0.69	0.50	4.05	−.10
EXPERIMENT 2						
Variable	20			0.05	4.10	0.10
Fixed	20			0.11	3.25	−.10
Random	20			0.11	3.18	0.07
EXPERIMENT 3						
Incongruent	20		0.35		3.20	0.31
Congruent	20		0.65		2.65	0.04

NOTE. Ghost Controls (GC/Computer Demonstration), Experiment 1: *No Attribution* = Children are only told to “Watch the computer,” *Agency Attribution* = Children are read a script describing the computer as animate, *Mechanical Attribution* = Children are read a script describing the computer as an inanimate artifact. Experiment 2: *Variable* = Border presentation occurs in a variable time interval, *Fixed* = Border presentation occurs in fixed time interval, *Random* = Sound accompanying border is variable. Social (Model Demonstration), Experiment 3: *Incongruent* = Model's touch and border presentation do not correspond, *Congruent* = Model's touch and border presentation correspond (see methods). None of the correlations reached statistical significance.

There were significant differences between conditions in first trial accuracy. As expected, performance in the Social condition was significantly better than in the Baseline condition (*Z* = −4.82, *p*<.001, Wilcoxan Signed Ranks Tests). Despite this result, there were no statistically significant differences in accuracy between the different GCs (**χ** *^2^*(2) = .59, *p* = .75, Kruskal-Wallis Test). There were also no statistically significant differences in performance between each of the different GCs and performance in the Social condition (Mechanical-Social: *Z* = −.82, *p* = .41; Agency-Social: *Z* = −1.27, *p* = .21; No Attribution-Social: *Z* = −.91, *p* = .37). However, only children in the No Attribution condition performed better in the GC than in Baseline (No Attribution-Baseline: *Z* = −2.83, *p*<.01; Mechanical-Baseline: *Z* = −.71, *p* = .48; Agency-Baseline: *Z* = −1.41, *p* = .16).

#### Age, Gender and Order Effects

There was a significant positive correlation between age in months (ranging from 48 to 59 months) and first trial accuracy in GC (*r* = .320, *p* = .02). Older children learned significant better than younger children in GC but not in either Baseline (*r* = .18, *p* = .18) or the Social condition (*r* = .08, *p* = .52). There was also an effect of gender in that girls outperformed boys with regards to first trial accuracy, but only in the Social learning condition (*r* = .34, *p* = . 01). Gender did not correlate with first trial accuracy in either Baseline (*r* = −.05, *p* = .74) or in GC (*r* = 11, *p* = .42). There were no significant order effects (*r*<.25, *p*>.05, Spearman **ρ**).

#### Survey Responses

All children attributed agency to the computer regardless of condition (c.f., Table 1). These attributions significantly differed from zero (*ts* (18)>7.00, *ps*<.001, One-Sample *t*-Test). As can be seen in Table 1, there were no significant correlations between agency attribution and first trial performance for either the Mechanical (*r* = −.10) or the No Attribution (*r* = .10) conditions (all *r*s<.1, all *p*s>.10). However, attributions made to the computer in the Agency Attribution (AA) condition did correlate more robustly with first trial accuracy (*r* = .44, *p* = .13). While not statistically significant, agency-attribution in the AA condition accounted for 20% of the variance.

Consistent with previous research, children in the present study learned in the GCs. Performance in GCs was significantly better than what was expected by chance alone. However, only the performance of children in the No Attribution condition was significantly better than Baseline. Children's performance in the GCs did not significantly differ from performance in the Social condition, where the model was an experimenter. All children learned from the model, with female children showing a slight advantage. Future research should address whether these gender differences accurately characterize learning in such computerized GCs. But, whereas all children spontaneously attributed agency and animacy to the computer, regardless of condition, these attributions did not significantly correlate with performance. This outcome is inconsistent with the Agency Attribution Hypothesis. There are several possibilities for this result. One possibility is that children failed to understand the script or the questions in the survey. A second possibility is that children learned from the GCs, regardless of condition because they all received training on the same computer prior to testing. This experience may have homogenized agency-attributions across conditions. Children's firsthand experience with the computer's affordances likely served as a scaffold for learning from the non-social cues provided by computer. To address these questions, Experiment 2 tested children on three different GCs without any scripts prior to testing. To minimize any firsthand experience, children were not familiarized with the computer or trained on the task prior to testing. Finally, children may have attributed agency/animacy to the computer across GCs because the agency/animacy cues provided by the computer were held constant throughout. To that end, Experiment 2 also manipulated agency cues provided by the computer.

In a series of studies with human infants, Biro & Leslie [38] manipulated various cues associated with the perception of agency including (A) *equifinal variation*- making contact with an object from different angles and directions, (B) *self-propelledness* or self-initiation of movement, (C) *action-effect*, where responses have specific consequences or outcomes (D) *combination* of A–C. Biro and Leslie (2007) reported that, while some cues led infants to attribute goals to inanimate objects, the presence of more than one of these cues led to robust agency attributions that did not differ from those made to a human hand in another study [37].

Following Biro and Leslie, we developed three different GCs that varied in agency and animacy cues:

- *Variable Border* (Variable), where a black border flashed around each item at a variable and accelerating rate, mimicking the rate of response of a human demonstrator. The appearance of the border corresponded with a chime.
- *Fixed Border* (Fixed), were the border flashed around each item in a fixed time interval along with a corresponding chime.
- *Random Sound* (Random), where the border and the corresponding chime did not coincide and were presented independently of each other.

As in Experiment 1, children were exposed to three trials where the computer acted as the model, highlighting picture items in the target serial order.

## Experiment 2

### Results and Discussion

#### First Trial Accuracy

Binomial tests were used to compare the probability of being correct on the very first trial of each of the three Ghost conditions to chance (*p* = .165). In contrast to Experiment 1, children failed to learn in any of the GCs (Variable: *p* = .26; Fixed: *p* = .26; Random: *p* = .66). There were no significant differences between first trial performance in the different GCs (**χ** *^2^*(2) = .466, *p*<.792, Kruskall-Wallis Test). Results are summarized in Table 1.

#### Age and Gender Effects

Given these results, we collapsed all three GCs together in order to assess relationships between age and gender. Results showed a significant positive correlation between age and first trial accuracy (*r* = .272, *p* = .039), indicating that the older children were more likely to learn in GCs than were younger children in our sample. However, in contrast to Exp. 1, gender did not correlate with first trial accuracy (*r* = −.056, *p* = .675, Spearman **ρ**).

#### Survey Responses

All children attributed agency to the computer regardless of condition (Table 1). These attributions significantly differed from zero (*ts* (18)>7.00, *ps*<.001, One-Sample *t*-Test). Children's agency attributions did not differ between GCs (*F*(2) = 1.66, *p* = .20, One-Way ANOVA). Additionally, Spearman's **ρ** correlation revealed no correlation between children's agency attributions and first trial performance (Table 1).

Together with Experiment 1 and previous work [25], [40] showing that children as young as three years of age can learn in GCs, the present study suggests that learning in these impoverished conditions depends on both task complexity and first-hand experience with the task. Hopper and colleagues [24] who have also employed different types of GCs varying in complexity have similarly argued that task complexity affects learning in GCs. However, here we go further and argue that in the absence of first-hand experience or familiarity with the affordances of tasks or objects such as tools, children fail to learn in GCs; including GCs varying in agency and animacy cues that, in other contexts, may scaffold learning [38]. This result fails to support a central prediction of the Agency Attribution Hypothesis given that, despite robust attributions of agency and animacy across conditions, children nonetheless failed to learn from the computer. Perhaps, children simply chose to ignore the computer, or are unable to remember what it has shown them, despite these attributions. As Laland [42] has argued, in the face of a difficult task or when uncertain, imitation should be the default social learning strategy. The fact that there was no evidence of imitation suggests a lack of competence.

The fact that children tested in similar tasks learned when provided with training (e.g., Exp. 1) but failed to learn without training (e.g., Exp. 2) demonstrates that the computer task lacks transparent or inherently meaningful affordances that buttress learning. It seems that without firsthand experience, children fail to understand the significance of the border; specifically, that the border serves as a cue for ordinality. Otherwise, children would have learned in Experiment 2 as well as in Experiment 1 and in earlier studies employing similar GCs [25]. Of course, it is possible that, without training, children will also fail to learn from an experimenter. A failure to learn from a model would suggest that the task is impossible to learn without direct, trial-and-error learning experience.

To address this question, Experiment 3 tested children in two social conditions: Congruent and Incongruent. In the Congruent condition, the experimenter's response and the computer's feedback corresponded. That is, when the experimenter touched a picture on the touch-screen a border flashed around that picture, and a chime accompanied each response. In the Incongruent condition, the experimenter's response and the computer's feedback did not correspond. That is, when the experimenter touched a picture on the touch-screen a border flashed around a different picture. Corresponding sounds such as a chime or a buzzer followed each response with a small delay. One might predict greater agency attribution in the incongruent condition where the computer appeared to act on its own, not in accordance with the experimenter's actions. As in Experiment 2, children were neither familiarized nor trained on the task prior to testing.

## Experiment 3

### Results and Discussion

#### First Trial Accuracy

Binomial tests were used to compare the probability of being correct on the first trial of each of the two social conditions to chance (*p* = .165). In contrast to Experiment 2, where children failed to learn in the various GCs, children learned in the Congruent condition (*p*<.001) and there was a trend toward learning in the Incongruent condition (*p* = .07). Performance in the Congruent condition was marginally better than performance in the Incongruent condition [*t* = −1.94, *p* = .06). Results are summarized in Table 1.

#### Age and Gender Effects

We evaluated age and gender effects for the Congruent and the Incongruent conditions separately. Results showed a significant correlation between age and first trial accuracy for both the Congruent (*r* = .480, *p* = .032) and the Incongruent conditions (*r* = .466, *p* = .038), indicating that the older children were more likely to learn in these Social conditions than younger children in our sample. As in Exp. 1, gender correlated with first trial accuracy, but only in the Congruent (*r* = −.524, *p* = .018) not the Incongruent condition (*r* = .314, *p* = .177, Spearman **ρ**). However, whereas girls outperformed boys in the Social condition of Experiment 1, boys outperformed girls in the Congruent condition in Experiment 3; a condition is equivalent to the Social condition in Experiment 1 except that there was no training. Thus, further testing is necessary to determine whether gender differences in imitation learning of this type are robust, or might be an artifact of relatively small sample sizes in the current study.

#### Survey Responses

As in Experiments 1 and 2, all children attributed agency to the computer regardless of condition (*ts* (18)>7.00, *ps*<.001, One-Sample *t*-Test). Children's agency attributions did not differ between the two conditions (*t* = .988, *p* = .33). Additionally, Spearman's **ρ** correlation revealed no relationship between children's agency attributions and first trial performance in these Social conditions (Table 1).

#### Comparison of Experiment 2 and Experiment 3

Given that there were no statistically significant differences in the performance of children in the various GC conditions of Exp. 1 and Exp. 2, we collapsed the groups in each Experiment in order to directly compare learning and agency-attributions in GCs (Exp. 2) versus Social (Exp. 3) conditions. Results showed that children in Social conditions learned significantly better on the first trial than children in GCs (**χ** *^2^*(1) = 21.557, *p*<.001, Kruskall-Wallis Test). Furthermore, there was a trend for children in the GCs to make more agency-attributions to the computer than children in the Social condition (**χ** *^2^*(1) = 3.068, *p* = .080, Kruskall-Wallis Test). When we excluded the Incongruent condition from the analysis (given that in this condition, the computer's feedback was independent of the feedback provided by the model), the differences in agency and animacy attribution between Experiment 2 and the Congruent (social) condition of Experiment 3 were significant, with children more likely to attribute agency in the GC than in the Social conditions (**χ** *^2^*(1) = 3.891, *p* = .049, Kruskall-Wallis Test).

In contrast to Experiment 2 where children failed to learn in the various GCs, children in Experiment 3 learned from the model despite the fact they did not receive any training prior to Testing. This result indicates that while training or familiarity with the computer task is necessary to learn in GCs using this computer paradigm, no such training is necessary if a human acts as the model. A direct comparison of the two studies confirms this conclusion, demonstrating that learning was significantly better in the Social conditions than in the GCs. This result is also consistent with other studies showing that learning in GC is impoverished relative to learning from a model when the task is of sufficient complexity [9].

The fact that children in the GCs (i.e., Exp. 2) were more likely to attribute agency and animacy to the computer than children in the Congruent (Social) condition (Exp. 3) is telling. This result suggests that agency attributions may play a role in learning in these conditions. One possibility is that, in the absence of a model, children's agency attributions increase motivation and attention to the computer and consequently, may result in learning in certain GCs, though, not all. This idea is consistent with recent research suggesting that even infants will infer a causal agent when they witness an event in the absence of an obvious actor [43].

Given how little information is present in GCs, it is impressive that young children learn in such conditions. The studies reported represent an attempt to understand how children learn under such impoverished conditions. Two possibilities were explored: the role of agency-attributions and the role of experience or familiarity with the task. The Agency Attribution Hypothesis [40], [44] for learning in GCs is inspired by research demonstrating that children, from infancy to adulthood, spontaneously attribute agency to objects that behave in a goal-directed fashion [33], [38], [45] and in certain circumstances such attributes lead to learning [38]. Along these lines, the Agency Attribution Hypothesis predicts that children learn in GCs, in part, because they attribute animacy and agency to objects that are self-directed and behave in a goal-directed fashion. Such cues lead to the perception of the object as a social agent. But it is also possible that direct, first-hand experience contributes to the understanding and attribution of intentions [46], [47] as well as to affordance learning [48], [49].

As predicted by the Agency Attribution Hypothesis, children across studies and experimental conditions attributed agency and animacy to the computer. Furthermore, children's attributions to the computer in the GCs of Experiment 2 and the Social conditions of Experiment 3 indicated that children in the GC were more likely to attribute agency and animacy to the computer than children in the Congruent (Social) condition, specifically. But, contrary to the Agency Attribution Hypothesis, these attributions neither correlated with performance nor significantly improved learning.

Experience with the task proved to be a better predictor of children's performance in GC than agency attributions. Consistent with previous work, we demonstrated that when provided with training, children learned a novel and entirely arbitrary 3-item serial rule from the computer in a GC. Without training, however, children failed to learn a similar rule in a GC. The same was not true when children were tested in a Social condition, where an experimenter served as the model. In Social conditions, children learned a 3-item rule on the very first trial, despite not being familiar with the task.

In addition to being animists [50], [51], children are also astute causal theorists using environmental cues to make accurate predictions about the physical world [52], [53], [54], [55]. For example, Gopnik and colleagues showed pre-school aged children that placing a toy atop a box resulted in a sound. But placing two toys did not produce a sound. These contingencies remained the same while different toys were placed atop the box. Children used this information to infer that only one toy (regardless of kind) produces a sound but two toys do not [56]. Such causal and inferential (i.e., abductive) reasoning mechanisms may have played a role in the present study and contributed to children's success in Experiment 1, for example. Note that this task is not unlike the computer task described here where asocial cues mediate learning. It is likely that a less complex version of the present computer task may result in learning and, it is also possible that in these less complex tasks, both causal (inferential) and social cognitive processes (mediating agency-attribution) interacted to facilitate children's learning.

Future research should explore the possibility that different systems, one mediating agency and another mediating physical causality, provide differential input to imitation learning mechanisms [28]. The prediction is that such a circuit would become active under impoverished learning conditions such as GCs or when encountering a novel problem involving complex tools such as a computer where either the model is absent or actions mediating specific results are opaque. Another fruitful avenue of research would compare learning in GCs that use this computer paradigm versus more traditional object-based tasks such as toys and other tools and assess whether agency-attributions differentially affect learning in these different paradigms. Understanding exactly how and why certain asocial conditions such as the GC promote vicarious learning while others do not is essential as more and more school districts adopt technology as teaching aides and, in some cases, as teacher substitutes.

## Experiment 1

### Materials and Methods

#### Ethics Statement

All children were recruited, trained and tested in the Smithsonian National Zoological Park by two trained research assistants who completed CITI training and were approved by the George Washington University (GWU) and Smithsonian Institution's (SI) Institutional Review Board (IRB). All training and testing for this study was specifically approved by GWU and SI IRB.

Legal guardians signed informed consent forms on behalf of the child under their care. Once the legal guardian read and signed the informed consent form, children were asked for their verbal assent prior to the start of Training and Testing. The consent and assent procedures used in this study were approved by the GWU and the SI IRB.

#### Participants

Fifty-four 4-year olds (mean = 53.42 months, SD = 3.95; Males = 26, Females = 28) were tested in the present study. Data from five children were excluded from the final analysis because they answered yes or no to all of the survey questions.

#### Simultaneous Chaining Task

In the simultaneous chaining task [12], [15], list items were displayed concurrently throughout each trial on a computer screen with a touch-sensitive screen and each item's position was varied randomly from trial to trial. The participant's task was to respond to each item in a particular order, regardless of its spatial position. Variation of spatial position prevents participants from performing the required sequence as a fixed–motor pattern or as a discrete set of responses to specific external spatial cues, such as the choice points of a maze. The variation of the spatial position of list items also eliminates the need for participants to form a representation of specific motor responses or to rely on a body schema to guide individual responses (cf. Figure 1).

The lists on which our participants were trained were composed of color photographs. These were presented to each participant on an iMac Apple Computer with a MagicTouch detachable screen. Photographs (1.5″×2″) were used as list items because they were easily discriminable and in plentiful supply. They were selected from a library created in our lab of more than 3000 digital images of natural and man-made objects (e.g., animals, people, scenery, flowers, cars, bridges, etc.).

#### Training

Participants were familiarized with the task prior testing. All children were introduced to a 3-item list of arbitrary photographs, appearing simultaneously on the touch-screen. With the aid of the experimenter, participants were encouraged to respond to all three pictures and to discover the correct sequence by trial and error. A response was defined as making contact with the touch-screen. Following a response, a border appeared around the picture, the computer generated a 1000 Hz tone and after a two second inter-trial-interval (ITI), the picture disappeared and re-appeared in a different spatial position. Reinforcement consisted of a brief “jumping man” [57] (a man doing a backward summersault accompanied by music or boisterous cheers and hand clapping). Training ended once participants responded correctly to a 3-item list of photographs on two consecutive trials without any assistance. Training took approximately 5 minutes.

#### Testing

All participants were first tested in two conditions: (a) baseline and (b) one of three randomly assigned ghost conditions (GC): (a) No Attribution, (b) Mechanical-Attribution, or (c) Agency-Attribution. Order of presentation was randomly determined and counterbalanced across participants in each treatment group. Finally, as a learning check on whether individual subjects who may not have performed accurately in the GC, were in fact capable of social learning, we presented a third social learning/imitation condition, where one of the experimenters demonstrated across three trials the correct order of the items on the screen. Including this condition allowed us to exclude the possibility that potential failures to learn in the GC condition were the product of a generally poor ability to learn by imitation. In order to demonstrate the advantage of the GC manipulation, it was important to show that individuals performed better in the GC than in baseline conditions. A within subjects design was required to serve these goals, and additionally increased our power to detect treatment differences. The main comparison of interest was that of learning in the GC condition between the three different treatments (No Attribution, Mechanical Attribution and Agency Attribution). We were primarily interested in whether children could learn in each of these GCs and whether enhancing their tendency to attribute agency to the computer facilitated learning. We did not create a fully counterbalanced treatment order design because we did not want the Social condition to influence the subsequent GCs were it to occur first. We wished the GCs to be uncontaminated by the observation of a human model demonstrating the correct order of the sequence, such that if participants attributed agency to the computer, they did so in the absence of having seen a human agent performing the task previously. We were additionally interested in interactions between the treatments and conditions – particularly whether children showed fewer differences between learning in GC and Social conditions in the Agency Attribution treatment, relative to the No Attribution and Mechanical Attribution treatments. We were not concerned with absolute differences between learning in GC and Social conditions as previous studies have demonstrated that, although learning occurs in GCs, learning in social learning conditions (i.e., with a human model) is stronger [25]. Note that any potential carry-over effects from the GCs to the Social condition due to presenting the Social condition last were not confounded by treatment. Thus, although the order of conditions were not completely counterbalanced, this does not present any particular problems of interpretation when considering the critical comparisons between performance in each of the GCs to the Social condition since the order of presentation (Ghost and then Social) was the same for each of the GCs. The same is true of any potential carry over effects from presenting baseline prior to the GCs for some children, as order was counterbalanced within treatment.

The procedures used in each condition were as follows:

- *Baseline*. In the baseline condition participants were not provided with any information as to the ordinal position of the pictures on the computer screen. At the start of the session, the laptop was placed in front of the child and the experimenters encouraged them to respond to the items on the screen. Participants had to discover the serial order of each item by trial and error. As a result, this condition served as a baseline measure of trial and error learning.
- *Ghost Control (GC)*. In this version of the GC, demonstrations consisted of the computer—acting as the model—automatically highlighting each item with a black border and chime in the correct serial order without any intervention by the human experimenter. After highlighting the last item in the sequence (Item C), ‘jumping man’ (the audio-visual reward) appeared and a new trial started after a 2 s inter-trial-interval. In order to discern the order of each list item, participants had to attend to the borders appearing around each item in the target serial order. From this, children had to infer (either explicitly or implicitly) that this event was functionally equivalent to a model's touch, or that the borders signified the correct sequence of responses, much like a human response did.
- *Social*. In the social condition, the child had an opportunity to learn the serial order of list items by observing the responses of the model during Demonstration. Demonstration began by one of the experimenters (“model”) saying ‘watch me’ and then proceeding to touch each picture on the screen in the correct sequence. Each of the model's responses was highlighted by audio and visual feedback from the computer (described below). This procedure was repeated three consecutive times. Following each correct demonstration trial, “jumping man” (audio-visual reinforcement) appeared and the model said, “Yay! I found, Jumping Man!” After the Demonstration period, a second experimenter reconfigured (<5 s) the computer used by the model and the child was allowed to make a response to the same list of photos.

Prior to testing in the Ghost Conditions, children were read one of the following scripts:

- *Agency Attribution*: “This computer is alive (Pets the computer). It likes to be tickled to play. When we tickle it, pictures appear. Let's watch the computer.”
- *Mechanical Attribution*: “This computer needs power (Shows power cord). We have to touch it to turn it on. When you touch it, pictures appear. Let's watch the computer.”
- *No Attribution*: “Watch the computer.”

Novel lists of arbitrary pictures were used in each condition. As a result, lists were never repeated within participants. Lists were randomly assigned to conditions and counterbalanced across participants such that each of three lists was used equally often in each of the three experimental conditions.

#### Survey

Following the completion of all Testing conditions, participants were given an oral questionnaire that assessed the degree to which they attributed agency and animacy to the computer. In total children were asked 9 Yes/No questions. ‘Yes’ responses corresponded with attributions of agency and were coded as 1. Questions 3, 6, and 8 were the exception. In these questions, ‘No’ responses were associated with attributions of agency and were coded as 1. This was done in order to control for “yes” biases regardless of agency attribution. The survey may be found in the Supporting Information (Survey S1).

### Data Collection and Analysis

The computer automatically recorded all responses. Our measure of learning was first trial accuracy. A trial consisted of a child's opportunity to respond to all three picture items displayed on the touch-screen. If the child responded to a picture out of order (e.g., A→C), this constituted an incorrect trial (0). If the child responded to all three items in the correct sequence, this constituted a correct trial (1). First trial accuracy assessed whether participants spontaneously—on the first trial—responded to all picture items on the touch-screen in the correct order without making any errors. This is the most sensitive measure of imitation because, after the first correct trial, it would be impossible to isolate what (if any) rule was learned from the model by cognitive imitation and what was learned by trial-and-error. For this reason we did not analyze number of trials to criterion. On a 3-item list the probability of a participant guessing the correct sequence on the first trial is 1/3 * 1/2 * 1/1 = 0.165.

## Experiment 2

### Materials and Methods

#### Ethics Statement

All children were recruited, trained and tested in the Smithsonian National Zoological Park by two trained research assistants who completed CITI training and were approved by the GWU and SI IRB. All training and testing for this study was specifically approved by GWU and SI IRB.

Legal guardians signed informed consent forms on behalf of the child under their care. Once the legal guardian read and signed the informed consent form, children were asked for their verbal assent prior to the start of Training and Testing for the study. The consent and assent procedures used in this study were approved by the GWU and the SI IRB.

#### Participants

Sixty 4-year olds (mean = 54.0 months, SD = 3.31; Males = 27, Females = 27) were tested in the present study. The data from seven additional children were excluded because they answered YES or NO to all of the survey questions.

Materials were the same as Experiment 1.

#### Testing

All participants were tested in one of three randomly assigned GC conditions (20 per condition):

- *Variable Border* (Variable): Within trials, a black border flashed around each item at a variable but accelerating rate, mimicking the rate of response of a human demonstrator. Borders were accompanied by a chime associated with a correct response.
- *Fixed Border* (Fixed): The border flashed around each item in a fixed time interval along with a corresponding chime.
- *Random Sound* (Random): The border flashed at an increasing rate as in the *Variable* condition but the sound corresponding with each border was incongruent, appearing independently of the flashing border.

All other aspects of the Testing procedures including the measures used and the administration of the survey were identical to those described above for Experiment 1.

## Experiment 3

### Materials and Methods

#### Ethics Statement

All children were recruited, trained and tested in the Smithsonian National Zoological Park by two trained research assistants who completed CITI training and were approved by the GWU and SI IRB. All training and testing for this study was specifically approved by GWU and SI IRB.

Legal guardians signed informed consent forms on behalf of the child under their care. Once the legal guardian read and signed the informed consent form, children were asked for their verbal assent prior to the start of Training and Testing for the study. The consent and assent procedures used in this study were approved by the GWU and the SI IRB.

#### Participants

Forty 4-year-olds (mean = 53.51 months, SD = 3.61; Males = 20, Females = 20) were tested in the present study. The data of six additional children were excluded from the final analysis because they answered YES or NO to all of the survey questions.

Materials were the same as Experiments 1–2.

#### Testing

All participants were tested in one of two randomly assigned social conditions:

- *Congruent*, the experimenter's response and the computer's feedback were congruent. That is, when the experimenter touched a picture on the touch-screen a border flashed around the picture. A chime accompanied each response.
- *Incongruent*, the experimenter's response and the computer's feedback were incongruent. That is, when the experimenter touched a picture on the touch-screen a border flashed around a different picture. Corresponding sounds including a chime and a buzzer accompanied responses with a delay.

All other aspects of the Testing procedures including the measures used and the administration of the survey were identical to those described above for Experiments 1–2.

## Supporting Information

Survey S1 **Agency Attribution Survey.** This survey was given to all children at the end of testing. The higher the score the higher the agency attribution.
(PDF)

## References

[1] Gopnik A, Glymour C, Sobel DM, Schulz LE, Kushnir T (2004). A theory of causal learning in children: causal maps and Bayes nets.. Psychological Review.

[2] Gopnik A, Sobel DM (2000). Detecting blickets: how young children use information about novel causal powers in categorization and induction.. Child Development.

[3] Caro TM, Hauser MD (1992). Is there teaching in nonhuman animals?. The Quarterly Review of Biology.

[4] d'Errico F, Vanhaeren M, Barton N, Bouzouggar A, Mienis H (2009). Out of Africa: modern human origins special feature: additional evidence on the use of personal ornaments in the Middle Paleolithic of North Africa.. Proceedings of the National Academy of Sciences of the United States of America.

[5] Williamson RA, Markman EM (2006). Precision of imitation as a function of preschoolers' understanding of the goal of the demonstration.. Developmental Psychology.

[6] Williamson RA, Meltzoff AN, Markman EM (2008). Prior experiences and perceived efficacy influence 3-year-olds' imitation.. Dev Psychol.

[7] Lyons DE, Pineda JA (2009). The Rational Continuum of Human Imitation.. Mirror Neuron Systems: The Role of Mirroring Processes in Social Cognition.

[8] Panger M, Brooks AS, Richmond B, Wood BA (2002). Older than the Oldowan? Rethinking the origins of hominin tool use? Rethinking the origins of hominin tool use.. Evolutionary Anthropology.

[9] Hopper LM (2010). ‘Ghost’ experiments and the dissection of social learning in humans and animals.. Biol Rev Camb Philos Soc.

[10] Thompson DE, Russell J (2004). The ghost condition: imitation versus emulation in young children's observational learning.. Developmental Psychology.

[11] Hopper LM, Spiteri A, Lambeth SP, Schapiro SJ, Horner V (2007). Experimental studies of traditions and underlying transmission processes in chimpanzees.. Animal Behaviour.

[12] Hopper LM, Lambeth SP, Schapiro SJ, Whiten A (2008). Observational learning in chimpanzees and children studied through ‘ghost’ conditions.. Proc Biol Sci.

[13] Huang CT, Heyes C, Charman T (2002). Infants' behavioral reenactment of “failed attempts”: exploring the roles of emulation learning, stimulus enhancement, and understanding of intentions.. Dev Psychol.

[14] Huang CT, Charman T (2005). Gradations of emulation learning in infants' imitation of actions on objects.. Journal of Experimental Child Psychology.

[15] Tennie C, Call J, Tomasello M (2006). Push or pull: Imitation versus emulation in human children and great apes.. Ethology.

[16] Csibra G, Gergely G (2009). Natural pedagogy.. Trends Cogn Sci.

[17] Call J, Carpenter M, Dautenhahn K, Nehaniv C (2002). Three Sources of Information in Social Learning.. Imitation in animals and artifacts.

[18] Heyes CM (2003). Four routes of cognitive evolution.. Psychological Review.

[19] Whiten A, McGuigan N, Marshall-Pescini S, Hopper LM (2009). Emulation, imitation, over-imitation and the scope of culture for child and chimpanzee.. Philos Trans R Soc Lond B Biol Sci.

[20] Zentall TR (2006). Imitation: definitions, evidence, and mechanisms.. Anim Cogn.

[21] Tomasello M, Call J (1997). Primate cognition.

[22] Beilock SL, Holt LE (2007). Embodied preference judgments: can likeability be driven by the motor system?. Psychol Sci.

[23] Yang SJ, Gallo DA, Beilock SL (2009). Embodied memory judgments: a case of motor fluency.. J Exp Psychol Learn Mem Cogn.

[24] Hopper LM, Flynn EM, Wood LAN, Whiten A (2010). Observational learning of tool use in children: Investigating cultural spread through diffusion chains and learning mechanisms through ghost displays.. Journal of Experimental Child Psychology.

[25] Subiaul F, Lurie H, Klein T, Holmes D, Terrace H (2007). Cognitive imitation in typically-developing 3- and 4-year olds and individuals with autism.. Cognitive Development.

[26] Byrne RW (2002). Imitation of novel complex actions: What does the evidence from animals mean?. Advances in the Study of Behavior Research Methods.

[27] Janik VM, Slater PJ (2000). The different roles of social learning in vocal communication.. Animal Behaviour.

[28] Subiaul F (2010). Dissecting the imitation faculty: the multiple imitation mechanisms (MIM) hypothesis.. Behavioural Processes.

[29] Carpenter M, Call J (2002). The chemistry of social learning.. Developmental Science.

[30] Tomasello M (1999). The Cultural Origins of Human Cognition.

[31] Scholl BJ, Tremoulet PD (2000). Perceptual causality and animacy.. Trends Cogn Sci.

[32] Biro S, Csibra G, Gergely G (2007). The role of behavioral cues in understanding goal-directed actions in infancy.. Progress in brain research.

[33] Premack D (1990). The infant's theory of self-propelled objects.. Cognition.

[34] Csibra G, Gergely G, Biro S, Koos O, Brockbank M (1999). Goal attribution without agency cues: the perception of ‘pure reason’ in infancy.. Cognition.

[35] Gergely G, Nadasdy Z, Csibra G, Biro S (1995). Taking the intentional stance at 12 months of age.. Cognition.

[36] Meltzoff AN (1995). Understanding the Intentions of Others: Re-Enactment of Intended Acts by 18-Month-Old Children.. Developmental Psychology.

[37] Woodward AL (1998). Infants selectively encode the goal object of an actor's reach.. Cognition.

[38] Biro S, Leslie AM (2007). Infants' perception of goal-directed actions: development through cue-based bootstrapping.. Dev Sci.

[39] Subiaul F, Cantlon JF, Holloway RL, Terrace HS (2004). Cognitive imitation in rhesus macaques.. Science.

[40] Subiaul F, Vonk J (2006). The Ghost in the Computer: Agency Attribution Mediates Cognitive Imitation in a Ghost Control.. PLoS One.

[41] Meltzoff AN (2007). ‘Like me’: a foundation for social cognition.. Dev Sci.

[42] Laland KN (2004). Social learning strategies.. Learning & behavior.

[43] Saxe R, Tenenbaum JB, Carey S (2005). Secret agents: inferences about hidden causes by 10- and 12-month-old infants.. Psychological science : a journal of the American Psychological Society/APS.

[44] Subiaul F, Romansky K, Cantlon JF, Klein T, Terrace H (2007). Cognitive imitation in 2-year-old children (Homo sapiens): a comparison with rhesus monkeys (Macaca mulatta).. Anim Cogn.

[45] Csbira G, Gergely G, Biro S, Koos O, Brockbank M (1999). Goal attribution without agency cues: the perception of ‘pure reason’ in infancy.. Cognition.

[46] Sommerville JA, Woodward AL (2005). Pulling out the intentional structure of action: the relation between action processing and action production in infancy.. Cognition.

[47] Sommerville JA, Woodward AL, Needham A (2005). Action experience alters 3-month-old infants' perception of others' actions.. Cognition.

[48] Tennie C, Call J, Tomasello M (2009). Ratcheting up the ratchet: on the evolution of cumulative culture.. Philosophical transactions of the Royal Society of London Series B, Biological sciences.

[49] Tennie C, Call J, Tomasello M (2010). Evidence for emulation in chimpanzees in social settings using the floating peanut task.. PLoS One.

[50] Carey S (1985). Conceptual Change in Childhood.

[51] Piaget J (1929). The Child's Conception of the World.

[52] Sobel DM, Munro S (2009). Domain generality and specificity in children's causal inference about ambiguous data.. Developmental Psychology.

[53] Gopnik A, Schulz LE (2004). Mechanisms of Theory Formation in Young Children.. Trends in Cognitive Science.

[54] Kushir T, Gopnik A (2005). Young children infer causal strength from probabilities and interventions.. Psychological Science.

[55] Schulz LE, Gopnik A, Glymour C (2007). Preschool children learn about causal structure from conditional interventions.. Developmental Science.

[56] Schulz LE, Gopnik A (2004). Causal learning across domains.. Developmental Psychology.

[57] Vonk J, Subiaul F (2009). Do chimpanzees know what others can and cannot do? Reasoning about ‘capability’.. Anim Cogn.

