# Supplementary material for: The Ghosts in the Computer: The Role of Agency and Animacy Attributions in “Ghost Controls”
Source: PLoS One. 2011 Nov 4;6(11):e26429. doi: 10.1371/journal.pone.0026429 (PMC3208540; doi:10.1371/journal.pone.0026429)
Supplement: Survey S1 — Agency Attribution Survey. This survey was given to all children at the end of testing. The higher the score the higher the agency attribution. (PDF) [file pone.0026429.s001.pdf]

GHOST CONTROL SURVEY

Participant ID: ___________________________

Question		
1. Does this computer know when it's right or wrong?	Y	N
2. Can this computer turn itself on?	Y	N
**3. *Does someone have to show this computer what to do?**	**Y**	**N**
4. Does this computer have feelings?	Y	N
5. Does this computer have a mind (point to your head)?	Y	N
**6. *Is this computer like other computers?**	**Y**	**N**
7. Can this computer be your friend?	Y	N
**8. *Is this computer just a machine?**	**Y**	**N**
9. Is this computer alive?	Y	N
TOTAL SCORE:	________	

____/__________________________

PI Name / Date

Instructions: Score all Ys as 1 and all Ns as 0. *Bolded should be scored in reverse (Y = 0, N = 1).
